# Supplementary material for: Are carbon emissions trading and green financial instruments synergistic? -Comprehensive quantitative research based on content analysis
Source: PLoS One. 2024 Mar 7;19(3):e0298601. doi: 10.1371/journal.pone.0298601 (PMC10919601; doi:10.1371/journal.pone.0298601)
Supplement: S1 Data — (ZIP) [file pone.0298601.s001.zip › Data/地级市Z_F工作报告.zip!/╔╜╢1⁄2/╝├─╧╩╨/└┤╫╘╣1⁄2╓┌║┼í╛┬φ┐╦╩2╛▌═°í┐_2012.docx]

各位代表：

　　现在，我代表市人民政府向大会报告工作，请予审议，并请各位政协委员和其他列席人员提出意见。

　　一、2008年以来的工作回顾

　　过去的四年，是济南发展史上很不平凡的四年。面对国内外复杂多变的经济环境，在中共济南市委坚强领导下，市政府与全市人民一起，以科学发展观为统领，按照“维护省城稳定、发展省会经济、建设美丽泉城”的总体思路，解放思想，提升境界，抢抓机遇，积极作为，卓有成效地开展工作，全市经济保持平稳较快发展。2011年，全市完成生产总值4406.3亿元，比2007年增长58%，人均64331元；实现地方财政一般预算收入325.4亿元，比2007年增长107.3%；社会消费品零售总额2023.1亿元，比2007年增长83.4%；固定资产投资1934.3亿元，比2007年增长106.2%。我们完成了市十四届人大一次会议以来的目标任务，实现了“十二五”时期良好开局。

　　——— 转方式调结构成效明显。把转方式调结构作为应对金融危机、提高经济发展质量和效益的重要举措。积极推进国家服务业综合改革试点工作，加快金融、信息服务、商贸物流、文化旅游和商务会展五大区域性服务业中心建设，服务业占生产总值比重达到53.1%，现代服务业占服务业比重达到46.9%，分别比2007年提高4.3个和5.5个百分点。金融支撑作用明显增强，2011年末人民币存贷款余额分别达到8275.8亿元和6893.7亿元，比2007年增长103.7%和87.4%。加大对工业新兴产业项目培育引进，实施重点技术改造项目计划，高新技术产业产值占规模以上工业总产值的比重达到38.66%，33个项目列入全省战略性新兴产业重点项目。大力增强创新驱动能力，举全市之力支持高新区加快发展，积极推进国家信息通信国际创新园、国家创新药物孵化基地、综合性国家高技术产业基地建设，济南云计算中心、中科院量子技术研究中心揭牌启用，国家千万亿次超级计算济南中心和全国首条高端集成电路封装测试生产线在我市建成运营，填补了国内空白。四年新增国家级工程技术研究中心和企业技术中心10家、省级114家，促进了创新发展。狠抓重点领域节能降耗，强化源头控制，淘汰落后产能，高污染和高耗能产业投资占比下降4.1个百分点，节能减排约束性指标全面完成。

　　——— 城市功能形象显著提升。紧紧抓住承办(举办)第十一届全运会、第七届园博会和十艺节的历史机遇，强力推进城市规划建设管理。四年来，累计投资2000亿元，城镇化率由63.05%提高到65.05%。完成新一轮城市总体规划，编竣重点规划设计600余项，实现中心城区控规全覆盖。“一城三区”建设加快推进，老城区棚户区、城中村、危旧简易楼改造成效显著，一批城市综合体建成开业，护城河通航和大明湖扩建改造工程全面完成。趵突泉连续8年喷涌，泉城特色更加凸显。东部新区“东荷西柳”一场三馆、省博物馆、省档案馆和一批总部经济楼宇落成投用，增添了奥体文博片区现代化气息。西部新区京沪高铁济南西站通车运行，省会文化艺术中心等重点工程加快建设，园博园、森林公园建成开放。滨河新区开发全面展开，小清河综合治理主体工程竣工。

城市功能不断完善，建设改造北园大街、二环东路高架及玉函路、奥体中路等市政道路，济莱、济菏、青银高速公路顺利通车。完成一批供水、供电、供气、供热和排水排污工程，城市公用设施覆盖率和服务管理水平有了新提高。完成腊山河、工商河、东泺河等13条河道治理，城市排水防洪能力增强。城市管理不断创新，强化数字化和网格化管理，城市环境综合整治有力推进，综合执法和管理水平明显提高，城管为民服务成效显著。整治破损山体108座，建成区人均公园绿地面积达到10.3平方米。抓好城区大气污染防治，市区空气环境质量改善率为9.4%。强化重点河流截污措施，列入全省考核的4条主要河流，均达到恢复鱼类生长的水质目标。

　　——— 统筹城乡发展扎实推进。全面落实强农惠农富农政策，新农村建设“十大行动”和统筹城乡“七大工程”取得显著成效。市级财政四年投入涉农资金77.3亿元，年均增长22.9%。现代农业加快发展，粮食生产连续9年丰收，2011年总产达到295.8万吨，蔬菜、畜牧、种苗、林果花卉四大产业不断发展，有机、绿色食品和无公害农产品认证达689个。农技推广体系逐步完善，农业机械化水平显著提高。农业龙头企业410家、专业合作社3278家，分别比2007年增加205家和2955家。农村基础设施建设持续加强，完成6座大中型水库和123座病险小型水库除险加固，治理水土流失面积351平方公里，完成南水北调东线济南段征地拆迁任务，东湖水库等工程建设顺利。创建国家森林城市初见成效，全市森林覆盖率达到31.1%。美国白蛾防治成效明显，秸秆综合利用率达到90%，农村沼气保有量19万户。基本实现村村通公路、客车、自来水、有线电视、互联网，完成38个小城镇提升改造和26.5万户农房建设任务，43处敬老院达到省级标准，全市过半乡镇、130万农村居民纳入城乡环卫一体化管理。扶贫工作成效显著，四年来，重点扶贫区域农民纯收入由人均4006元提高到7550元，年均增长17.2%，高于全市农民纯收入增幅3.9个百分点。县区经济发展加快，帮扶商河成效显著，章丘在全国县域经济百强中位次前移，2011年，4个县(市)区和高新区地方财政一般预算收入超过20亿元。

　　——— 城乡居民生活持续改善。四年来，承诺的43件民生实事全部兑现。2011年，市本级用于民生和社会领域支出占全部地方财政预算支出的54.8%，比2007年提高4个百分点。城市居民人均可支配收入28892元、农民人均纯收入10412元，比2007年分别增长60.5%和65.3%。居民消费价格指数涨幅为5.4%，启动社会救助和保障标准与物价上涨挂钩联动机制，缓解物价上涨对低收入居民基本生活的影响。积极促进就业，四年累计新增城镇就业54万人，农业富余劳动力转移就业57.2万人次，历年城镇登记失业率控制在4%以内。城乡居民社会养老保险实现全覆盖，企业离退休人员人均月养老金由2007年1090元提高到1825元，城镇居民基本医疗保险全面推开，新型农村合作医疗政府补助标准提高到年人均200元，实现应参尽参。城市低保月人均标准提高到400元，农村低保标准提高到每人每年不低于1800元。启动38个集中片区和35个零星片区棚户区改造。四年累计开工建设保障性住房7.49万套，其中公共租赁住房2.32万套、经济适用房1.68万套、棚改安置房3万套、廉租住房4853套，向6330户市民发放廉租住房补贴6430.5万元。市区人均住房建筑面积30.3平方米，比2007年增加2.4平方米，群众居住生活条件得到改善。

　　——— 各项社会事业全面进步。大力实施人才强市战略，启动“齐鲁人才特区”建设，完成“5150引才计划”，引进高层次创新创业人才210人。实施《济南市中长期教育改革与发展规划纲要》，四年投入教育经费185亿元，改造重建和新建学校636所，新建公办幼儿园50所，确定外来务工人员子女定点学校56所，城乡义务教育经费保障机制全面落实。完成2366个村级卫生室标准化建设，在全省率先建成基层卫生服务体系。开工新建市图书馆、群众艺术馆、美术馆，基本建成四级公共文化服务体系，推出大型电视片《天下泉城》、京剧《重瞳项羽》和《李清照》等一批文化精品，“泉城大舞台”等群众文化活动蓬勃开展。成功举办首届中国非物质文化遗产博览会、第十九届全国图书博览会、第五届中国京剧节。圆满完成第十一届全运会济南主赛区和第七届园博会承办工作，提高了我市的知名度和美誉度。我市运动员奋力拼搏，在国内外赛事中获得金牌243枚。计划生育工作走在全省前列，2011年全市人口自然增长率为4.34‰。加强民族团结和宗教工作，支持慈善事业发展。社会科学、档案史志、广播电视、妇女儿童、老龄、残疾人、气象、防震减灾等工作不断加强。

　　——— 改革开放取得新成绩。积极推进重点领域和关键环节改革。出台加快建设文化强市的实施意见，完成经营性文化事业单位转企改制。城市投融资平台改革重组为四大投资集团公司，市、县、镇(乡)机构改革顺利完成。建立市、县、镇、村行政审批便民服务体系，精简行政审批278项。公共资源交易中心建成运行，网上政府建设不断加强，12345市民服务热线在服务群众、畅通民意方面发挥了纽带作用，受到广大群众的欢迎。突出抓好医药卫生体制五项改革，实现政府办基层医疗卫生机构基本药物制度全覆盖，每门诊人次和每住院床日药费分别下降24.6%和29.3%。促进竞争性领域国有资产重组，监管企业资产结构和运营质量明显改善。出台水利改革实施意见，集体林权主体改革基本完成，农村土地承包经营权流转2.74万公顷。加快开放型经济转型升级。2011年实现外贸进出口总额104亿美元，其中出口60.5亿美元，比2007年分别增长67.3%和76.1%；实际到账外资11亿美元，完成对外承包工程21.6亿美元，比2007年分别增长96.2%和155%；服务外包4.5亿美元，年均增长60%，占全省比重40%以上。口岸设施功能不断完善，友好城市合作有了新发展，外事、侨务、对台工作取得新成绩。

　　——— 民主法治建设不断加强。深入推进依法治市，坚持依法行政和政务公开，认真执行人大及其常委会的决议、决定，依法接受人大监督，支持政协发挥政治协商、民主监督、参政议政的职能作用，自觉接受群众和社会舆论监督，四年受理人大代表议案建议1476件、政协提案2187件，均落实办复。积极开展“下基层、解难题、办实事”、“执政为民、廉洁高效”和“创先争优”活动，启动行政执法电子监察和行政复议试点，健全反腐倡廉和科技防腐机制，加强审计监督，深化源头防治腐败各项工作，促进了机关作风转变和行政效能提升。创新社会管理，化解社会矛盾，基层基础建设、社区管理和信访工作得到加强，健全社会治安综合治理和防控体系，依法打击各类违法犯罪活动，促进了社会平安和谐稳定。强化工商管理服务和安全生产、产品质量、食品药品监管，进一步规范了生产和市场秩序。广泛开展“迎全运、讲文明、树新风”等精神文明创建活动，涌现出贴心民政、阳光大姐、泉城义工等知名品牌和全国著名的道德模范标兵人物。国防动员、民兵预备役建设、人民防空、双拥共建和优抚安置工作成绩显著。圆满完成四川省北川县擂鼓镇及县城灾后重建和对口援藏、援疆、帮扶重庆各项任务。

　　各位代表！四年来的发展变化，是省委、省政府和市委正确领导的结果，是全市人民艰苦奋斗、开拓进取的结果，是省会各界团结一致、共同努力的结果。在此，我代表市人民政府，向全市各族人民，各级人大代表、政协委员、各民主党派、工商联、无党派人士、各人民团体和各界人士，向驻济人民解放军、武警官兵和中央、省驻济单位，向港澳台同胞、海外侨胞和国际友人，表示崇高的敬意和诚挚的感谢！

　　各位代表，四年的实践使我们深刻体会到，做好济南的工作，必须坚持以科学发展观统领全局，牢牢把握发展第一要务不动摇，突出好字当头，毫不放松地推进经济社会又好又快发展；必须坚持以转变经济发展方式为主线，通过结构调整和自主创新培育新的增长点，加快省会经济转型升级步伐；必须坚持抓城建就是抓发展、惠民生，把城市建设作为推动经济社会发展的载体和动力；必须坚持深化改革、扩大开放，用改革、创新、开放的思路和办法，破除思想观念和体制机制障碍，增强科学发展的活力；必须坚持以人为本、执政为民，注重社会公平和公共资源均衡配置，使发展成果惠及广大人民群众；必须坚持解放思想，与时俱进，抢抓机遇，真抓实干，把提升境界标准与增强执政能力结合起来，努力建设务实、高效、廉洁的服务型政府。

　　各位代表，四年来的成绩令人鼓舞，但我们清醒地看到，面临的困难和问题仍然不少。综合实力与竞争能力还不够强，实体经济支撑和内需拉动作用比较薄弱，对我市生产总值、服务业增加值和实际到账外资指标的完成造成一定影响；省会优势和泉城特色发挥得还不够充分，城市综合服务功能、承载力和辐射力亟待提升；县域经济发展相对滞后，园区经济和特色产业还不强；对外开放程度还不高，体制机制活力不足，办事效率、创新能力不能很好满足科学发展需要；社会管理基层基础工作相对薄弱，公共服务、保障改善民生和维护社会稳定等方面还有一些问题需要解决；政府自身建设水平需要进一步提高，转变作风、提高效能的任务相当繁重。尤其需要注意的是，在有些干部中不同程度地存在着精神懈怠、能力不足、脱离群众的问题，腐败现象仍有发生。对这些问题，我们必须高度重视，采取有力措施切实加以解决。

　　二、今后五年工作的基本思路和主要目标任务

　　今后五年，是济南率先建成更高水平小康社会、开启现代化建设新征程的关键时期，也是迎接挑战、大有作为的战略机遇期。当前，国内外经济形势复杂多变，社会矛盾凸显，发展中不稳定、不协调、不可持续的问题仍然突出，前进道路上充满风险和挑战。但是从总体看，机遇大于挑战，国内外发展的有利条件大于不利因素，工业化、信息化、城镇化、市场化、国际化融合发展的趋势没有改变，这为我市长期向好发展提供了有利环境和诸多机遇。我市经过多年的改革发展，综合实力增强，功能形象提升，社会事业进步，为今后平稳较快发展奠定了坚实基础。刚刚召开的中国共产党济南市第十次代表大会，回应人民群众的新期待，规划了未来五年省会经济社会发展的宏伟蓝图，进一步明确了目标任务，有力激发全市人民奋斗热情，大大增强我们做好各项工作的信心。

　　今后五年政府工作的基本思路是：高举中国特色社会主义伟大旗帜，以邓小平理论和“三个代表”重要思想为指导，深入贯彻落实科学发展观，牢牢把握科学发展主题和加快转变经济发展方式主线，按照中共济南市第十次代表大会的部署要求，以群众为根本，以实践为标准，发挥省会优势，建设美丽泉城，大力实施新型城市化、新型工业化、创新驱动、富民惠民战略，打造实力济南、活力济南、魅力济南，统筹推进经济建设、政治建设、文化建设、社会建设和生态文明建设，努力在全省率先建成更高水平的小康社会，奋力开启现代化建设的新征程。

　　根据“十二五”规划，着眼今后五年，工作中要充分体现“发展更好、城市更靓、管理更优、生活更美”的要求。总的目标任务是：

　　——— 全面增强综合经济实力。牢牢把握发展这个第一要务，坚持稳中求进、好中求快，保持经济平稳较快增长。全市生产总值年均增长11%，地方财政一般预算收入年均增长13%，固定资产投资年均增长18%以上，力争主要发展指标在全国同类城市位次前移。做大做强实体经济。坚持服务业和工业双轮驱动，大力推进金融、信息服务、商贸物流、文化旅游和商务会展五大区域性服务业中心建设，围绕传统优势产业和战略性新兴产业发展，加快培育汽车、信息、机械装备、新能源及节能环保、石化及新材料、食品医药、轨道交通装备等7个千亿级产业集群，着力打造一批过百亿企业，增创发展新优势。分类推进县区经济跨越发展。推动历下、市中、槐荫、天桥四个中心区产业升级，全力打造现代服务业聚集发展先行区；鼓励历城区、章丘市跨越提升，加快城市建设和产业集聚发展，争当济南跨越发展新的增长极；推动落实帮扶商河发展和三年突破平阴的政策措施，支持济阳加快建设产业北跨发展区，推动长清加快融入中心城区发展。各发展主体都要按照增幅高于全市平均水平的要求确定目标任务，通过五年努力，实现中心区率先跨越、近郊区争先进位、远郊区迅速崛起。强化园区建设。支持高新区率先发展，尽快进入全国一流行列。加快8个省级开发区建设发展，力争在全省开发区竞相发展中位次不断前移。提高济南在省会城市群经济圈中的首位度，加快建设具有较强辐射带动能力的现代化省会城市和区域中心城市。

　　——— 加快转变经济发展方式。突出“主题主线”，加快省会转型升级步伐。着力优化需求结构。以项目建设为抓手，狠抓增量消费和招商引资，调整优化投资结构，把增投资与扩消费更好地结合起来，构建扩内需长效机制，加快形成以内生增长为主的消费、投资、出口协调拉动新格局。推动产业优化升级。巩固提高农业，大力发展都市型、城郊型现代农业；做大做强工业，改造提升传统工业，大力发展高端高质高效产业；加快提升服务业，优先集聚发展现代服务业。促进三次产业协调竞相发展，构筑服务经济主体带动、高新技术产业和先进制造业强力支撑、现代农业扎实推进的现代产业体系。强化创新驱动。深入实施科教兴市、人才强市和质量兴市战略，发挥省会优势，整合创新资源，培育创新主体，全面提高自主创新能力，集中力量攻克一批关键核心技术，培育一批创新型企业和领军人物，争创更多的知名品牌、驰名商标和国家标准，率先建成国家创新型城市。提升经济增长效益。注重项目前期策划研究，强化投入产出分析，力争用较少的要素投入，获取较大的经济和社会效益。提高绿色发展水平。重视发展低碳经济和循环经济，强化节能减排，压缩高耗能、高污染和产能过剩行业，坚持节约集约用地，努力构建资源节约型和环境友好型社会。

　　——— 努力建设美丽泉城。全面提高城市规划建设管理科学化水平。强化规划引领作用，精心组织总体规划的深化完善和重点区域、重要节点的城市设计，以高水平规划促进城市高标准建设、高效能管理。加快“一城三区”开发建设，老城区继续推进棚户区、城中村和危旧楼群改造，促进传统工业企业有序外迁、转型升级，改造提升低档次物流和专业批发市场，基本建成东部新区、西部新区核心区、滨河新区核心区和重点功能区。积极推进北跨战略，加大规划建设力度，以交通设施、产业园区、生态旅游、强镇建设为依托，加快形成北跨区域发展新格局。实施积极的南部山区保护与发展战略，科学有序地发展南部经济，优化生态环境，提高当地居民生活水平。完善功能互补、布局合理的城镇体系，章丘、济阳、平阴、商河要以建设济南次中心城市为目标，高起点规划建设。努力打造区域绿色发展新高地，加快省会城市群经济圈发展，主动融入“一黄一蓝”国家战略，在更高层次上拓展发展空间。进一步加强城市基础设施建设，重点抓好城市道路和公共停车场建设，优化路网结构和交通体系，加快城市轨道交通和城际铁路规划建设，优先发展公共交通，率先建成“公交都市”。提高交通管理科学化水平，有效缓解城市交通拥堵状况；提高城市净化、绿化、亮化、美化水平，改善城市景观形象；提高供水、供电、供气、供热保障水平，方便人民群众生活；提高城市污水处理、垃圾处理和大气治理水平，创造宜人居住环境；提高消防、人防、防震、防洪、气象设施水平，确保城市防灾安全。进一步创新城市管理，深入推进环境综合整治，不断完善长效管理机制，提升“人民城管”服务品牌。突出泉城特色，实现泉水持续喷涌，推进泉水申遗、先观后用、深度开发，切实提高饮用水质量。经过五年努力，城市功能和现代化水平显著提高，城镇化率达到70%左右，国家生态城市和国家森林城市基本建成，让美丽泉城天更蓝、水更清、空气更清新、绿化更多彩。

　　——— 深入推进改革开放。坚持社会主义市场经济的改革方向，推进重点领域和关键环节改革。着力解决全局性、深层次、体制性矛盾，不断增强发展活力，促进社会和谐。深化医药卫生体制改革，健全基本医疗卫生制度和基本药物制度。推进文化体制改革，促进文化大发展大繁荣。推进行政管理体制改革，加强法治政府和服务型政府建设。推进财政体制改革，建立完善公共财政体制。推进市四大投资集团改革，增强融资能力和开发实力。深化国有企业改革，加快国有经济战略性调整。鼓励支持非公有制经济加快发展，激发全民创业创新活力。推进农村经营、土地管理、金融服务、流通体制改革，引导发展集体经济。全面提高对外开放水平。大力实施开放带动战略，坚持高质量“引进来”，优化外资结构，吸引更多的市外企业和资金向济南聚集，构建全方位、多层次、宽领域的利用外资新格局。加快转变外贸增长方式，优化出口结构，推动加工贸易转型升级，大力发展服务贸易，增强口岸服务功能，保持进出口稳定增长。积极稳妥“走出去”，支持企业到国外开拓市场、扩大贸易。坚持引资与“引智”相结合，积极引进先进技术、管理经验、高效机制和高素质人才，带动先进发展理念在我市推广传播。适时建设一批国际教育、医疗、文化设施和生活居住服务区，努力营造省会国际化氛围。

　　——— 大力推进文化强市建设。以全力办好第十届中国艺术节为契机，加强公共文化服务体系建设，建设一批市级公共文化设施和基层文化基础设施，加快构建覆盖城乡的四级公共文化服务网络，强化公益性、均等性文化服务，推动公共文化资源更多地向社区和农村倾斜。采取市场化办法，动员社会力量建设100所不同门类、各具特色的博物馆。加大四大泉群、三大名胜、百年商埠、千年古城的系统整合和保护，进一步提升“天下泉城”、“名士济南”文化品牌影响力。整合壮大文化人才队伍，创作一批在全国具有影响力的文化精品。大力发展文化产业，深入挖掘传统历史文化资源，培育现代文化产业项目，打造一批特色文化产品、知名文化企业和文化产业集聚区。创新文化发展体制机制，深化国有文化单位改革。培育“诚信、创新、和谐”的济南城市精神，提高全社会思想道德素质、科学文化素质和城市公共文明程度，争取跻身全国文明城市行列。

　　——— 切实增强人民群众幸福感。坚持以人为本、民生优先，努力让广大市民生活得更加美好。健全收入稳定增长机制，提高居民工资性、经营性、财产性收入，突出做好农民、企业职工、中低收入者和困难家庭增收工作，城市居民人均可支配收入年均增长11%以上，农民人均纯收入增幅高于城市居民收入增幅，有效控制城乡收入差距扩大趋势。实施更加积极的就业政策，多渠道开发就业岗位，完善市场就业机制，支持高校毕业生自主创业，以创业带就业、促增收，努力保持就业形势基本稳定。建立更高水平社会保障体系，实现城乡基本养老、基本医疗保障制度全覆盖，完善城乡困难群众社会救助体系，建立城乡低保标准自然增长机制，基本社会保险覆盖率达90%以上。优先发展教育，推进各级各类教育均衡发展，强化措施缓解入托难问题，逐步缩小区域、校际办学条件和教学水平差距。加快发展医疗卫生事业，改善基层医疗卫生条件，有效缓解人民群众看病难看病贵问题。着力解决城乡中低收入家庭住房困难，新建保障性住房7.6万套，城镇人均住房面积达到33平方米。加强安全生产和食品药品监管工作。社会管理富有活力，为民服务机制更加健全，平安建设持续深入，人民合法权益得到切实保障，社会更加公平公正、和谐稳定。群众安全感、健康水平、生活质量均有较大提高，人民生活得更加幸福美满。

　　三、全面做好开局之年的工作

　　今年是新一届政府开局之年，也是实施“十二五”规划承上启下的关键一年。做好今年工作，必须全面贯彻中央“稳中求进”的工作总基调，按照“四个牢牢把握”和“四个着力”的基本要求，以创新驱动、提质增效、统筹发展为着力点，正确处理保持经济平稳较快发展、调整经济结构和管理通胀预期三者关系，统筹推进稳增长、控物价、调结构、拓空间、抓改革、促开放、惠民生、保稳定各项工作。经济社会发展主要预期目标是：生产总值增长10.5%，地方财政一般预算收入增长15%，固定资产投资增长18%，社会消费品零售总额增长15%。服务业增加值增长11%，规模以上工业增加值增长12%，高新技术产业产值比重提高1个百分点。外贸出口增长11%，实际到账外资增长11%。城市居民人均可支配收入、农民人均纯收入均增长11%，居民消费价格总水平涨幅控制在4%左右，城镇登记失业率控制在4%以内，人口自然增长率控制在4.7‰以内。完成省政府下达的年度节能减排目标任务。工作中努力争取各项指标完成得更好。

　　(一)保持经济平稳较快发展。全面落实国家宏观调控政策，增强经济发展的内生性、协调性。一是扩大消费需求。提高居民消费能力，健全职工工资正常增长和支付保障机制，继续提高企业最低工资标准，合理增加城乡居民特别是低收入群众收入。拓展消费领域，优化消费结构，加快生存型、温饱型消费向小康型消费转变，从以物质消费为主向物质、文化消费并重转变，不断提高精神文化消费的比重。积极扩大增量消费，打造文化、旅游、商务等消费热点，落实结构性减税政策，用优良的购物环境、旅游环境和服务环境，吸引国内外消费群体来济消费。健全商贸流通体系，完善大型商贸综合体配套服务，加快农产品物流交易中心、“社区便民放心菜”、“放心早餐”工程建设，继续推进“万村千乡”及“农超对接”工作。落实全国肉菜追溯、家政服务体系建设试点城市任务。二是促进投资稳步增长。扎实搞好招商引资，推动社会力量投资办实业，全力抓好项目建设。要“抓大不忘小”，盯住世界500强和国内大企业，多引进一些符合转方式调结构的现代农业、新型工业和现代服务业的大项目、好项目，同时要大力支持成长性好、效益好的中小项目发展。要“抓新不忘老”，既要抓好新项目的引进和建设，也要重视老企业转型升级，通过装备更新和技术改造，使老企业焕发新的生机和活力。要“抓外不忘内”，既要抓好外资项目和市外引进项目，更要鼓励市内企业家和有志之士干事创业、多上项目。要“抓快不忘好”，既要重视投资规模的扩张，也要关注投资质量、结构和效益，加大高端高质高效新兴产业的投资。转变工作思路，创新服务机制，由主要依靠政策招商转为更多地依靠优化环境、优质服务和优势资源招商。大力实施“项目建设三年行动计划”，强化各级领导干部包项目责任制，对好项目、大项目全程跟踪服务，完善重点项目分级联系和绿色通道制度。充分发挥工商联和各类商会的作用，营造民营企业公平参与竞争的市场环境，促进民营经济扩大投资、做大做强。加强高效财源建设，提高税收征管水平。组织好对各县(市)区、高新区项目和园区建设现场观摩与考核评先。三是保持物价和市场稳定。加强主要农产品、基本生活必需品、重要生产资料的供应，搞好产运销衔接，降低流通成本。稳妥推进政府管理价格调整，完善社会救助、保障标准与物价上涨挂钩联动机制，健全价格监测预警体系，严厉查处发布虚假信息、囤积居奇、操纵价格等非法行为，打击商业欺诈、制假售假行为，保持良好的消费环境和市场秩序。

　　(二)推动服务业优化提升。把推动服务业发展作为产业结构调整的战略重点，促进服务业与工业融合发展、提速升级。一是加快优势产业发展。深化国家服务业综合改革试点，加快五大区域性服务业中心建设。金融服务业重点是积极引进外资和外埠银行，大力发展地方金融保险机构和证券期货市场，组建新型金融机构，促进企业上市和资本市场发展，加快建设区域性金融中心。信息服务业重点是推进“三网融合”，突出发展软件、服务外包、动漫游戏、物联网等产业，推动支柱产业高端化。商贸物流业重点是加快修订物流产业规划，加快物流园区、中心、节点三级网络建设，培育大型第三方物流企业。文化旅游业重点是加快发展文化创意、现代演艺、移动多媒体等新兴文化产业，加大旅游资源整合开发，拓展观光、体验、休闲、度假旅游，推广“天下泉城”品牌，打造国内外知名的旅游目的地和集散地。商务会展业重点是加快发展财务、法律、咨询和市场交易等商务服务业，办好第六届信博会，引进会展品牌，培育大型会展企业。房地产业重点是落实国家房地产调控政策，合理调整住房供应结构，抑制投资投机性需求，加强对物业公司的监管，促进房地产市场平稳健康发展。住宿餐饮业重点是加快经营结构和业态调整，改造提升基础设施，提高服务质量和品位。鼓励支持阳光大姐等一批企业拓宽领域、优化服务，大力发展家庭服务、社区服务、养老服务、健康服务等生活性服务业。二是加快服务业载体建设。推进20个重点服务业集聚区建设，重点启动东部金融新区规划建设，提升金融商务中心区，加快中国软件名城、智慧泉城、动漫泉城和服务外包基地建设，促进商业核心区、商业中心区和10个特色街(园)区繁荣发展，改造提升部分传统批发市场，筹建西客站会展场馆。高起点规划建设一批产业特色鲜明、吸引力强的城市综合体。三是加强政策支持。落实国家服务业综合改革试点实施方案和加快服务业跨越发展的若干政策，在规划、土地、财税、金融等方面增强对服务业发展的支持力度，吸引更多社会资金投向服务业。鼓励制造企业剥离生产性服务业，推进政府部门、事业单位中间服务环节的改造和剥离，营造有利于服务业发展的体制和政策环境。

　　(三)加快工业跨越发展。把工业作为发展实体经济的重要支撑，大力提高整体实力。一是科学规划布局。加强工业发展战略研究，按照突出特色、优势互补、错位发展、全面提升的原则，大力推进新型工业化，强化综合措施，鼓励老城区传统工业企业向外部工业区转移。优化“一高三区”工业布局。高新区：以中心区、孙村片区、出口加工区为依托，以交通装备、电子信息、食品药品、机械装备等产业为重点，打造高新技术产业密集区和先进制造业集聚区。东部工业区：以明水开发区、临港开发区和济南航空产业园为依托，以新能源、汽车制造、生物化工、飞机维修等产业为重点，打造跨越发展新的增长极。西部工业区：以济南经济开发区、平阴工业园和槐荫工业园为依托，以机械装备、水泥建材、清洁能源、家用电器和农副产品深加工等产业为重点，打造突破发展的产业集聚区。北部工业区：以济南化工产业园、济北开发区和商河经济开发区为依托，以精细化工、食品饮料、纺织服装等产业为重点，打造北跨发展特色产业基地。二是促进产业扩规提效。围绕做大做强传统优势产业、培育壮大战略性新兴产业，实施百项创新升级重点项目，抓住技术改造、项目引进、兼并重组等重要环节，推动重汽、浪潮等龙头企业和产业链上的关键企业在本地配套和招商拓展，支持山水、二机床、力诺、齐鲁制药、九阳家电等重点企业做大做强。加快33个省重点战略性新兴产业项目建设，抓好圣泉生物质材料、天岳碳化硅衬底材料、红帆直线电机等一批具有自主知识产权的重点项目，实施新能源汽车、基因工程药物、集成电路、光伏材料、云计算基础装备等产业创新发展工程。加强对重点产业集群、大型骨干企业及煤电油气运等生产要素的调度、监测和服务，全力保障工业运行。推进企业管理创新和技术改造，加强质量、标准和品牌建设，不断挖潜增效，提高生产经营效益。三是提高园区集聚发展水平。实施产业基地公共服务能力提升工程，提高管理效率和服务水平，增强承载能力，吸引企业和项目向园区集中，尽快拉长产业链，形成产业集群。全面启动综合性国家高技术产业基地建设，抓好浪潮光电子产业园、信息通信产业园、福瑞达生物医药产业园等项目建设，全力支持高新区争创一流，支持8个省级开发区争先进位。四是扶持中小企业加快发展。开展“中小企业服务年”活动，完善融资担保、创业辅导、技术创新、信息化、服务中心等平台，支持小微企业做专做精，培育一批技术先进、成长性强、效益好的优质企业快速成长，形成数量庞大的企业群体。五是推进信息化和工业化深度融合。继续开展“两化融合”试验市建设，健全工业软件推广应用机制，围绕技术改造、研发设计、新型业态培育，加快实施三网融合、下一代互联网、物联网等应用示范工程，促进新一代信息技术与工业融合渗透，打造“两化融合”示范区和10个示范企业。

　　(四)统筹城乡协调发展。始终把“三农”工作放在重要位置，着力推进农业科技创新，促进农业增产、农民增收。一是大力发展都市型、城郊型农业。加快推进环城都市农业圈、南部山区生态观光农业带、中部平原特色精品农业带、黄河以北设施高效农业带和50个特色品牌基地、200个都市农业园区建设。抓好3亿公斤粮食增产规划和蔬菜、畜牧、种苗、林果花卉四大产业振兴规划，力争粮食连续十年丰收。认真落实中央1号文件精神，加强农业科技创新，改善农业设施和装备条件，提高农产品生产标准化水平。加快现代农业示范区建设，新认定市级农业龙头企业50家以上、合作社示范社100家。二是加强以水利为重点的农村基础设施建设。实施狼猫山等11座中小型水库和139座塘坝除险加固工程，推进玉符河、北大沙河等河道治理和一批防汛应急工程。新增旱涝保收面积1.33万公顷，发展节水灌溉面积2万公顷，推进南水北调配套工程建设。新增造林面积1.3万公顷，全市森林覆盖率增长1个百分点以上。

　　全面完成无规定动物疫病区建设任务，加快气象防灾减灾工程建设。加强农村路、电等基本公共事业建设，再解决19.5万人饮水安全问题。三是多渠道增加农民收入。培育壮大农产品流通中介组织和农民经纪人队伍，推进农产品直销，扩大农超、农校、农企产销衔接，增加农民经营性收入，引导农民就地就近转移就业。全面落实中央扶贫开发会议精神和新十年扶贫开发纲要，实现6万贫困人口人均增收1100元。四是加快推进城镇和新农村建设。支持具备条件的中心镇向小城市发展，培育发展一批工业强镇、商贸重镇和旅游名镇。按照科学规划、适度集中、注重功能、保证质量、因地制宜、分类指导的原则，加快农村新型社区建设。继续实施城乡环卫一体化，加强农村环境综合整治，着力解决部分地区脏乱差现象。

　　(五)提升城市规划建设管理水平。抓好重点项目，推进生态建设，提升内涵品位，增强城市承载功能。一是加快“一城三区”规划建设。完善科学规划体系，高标准搞好核心区的城市设计，进一步优化城市功能。老城区：实施大明湖(护城河)与小清河通航、明府城、老商埠区保护改造工程，启动千佛山风景区改造提升工程，继续抓好棚户区、城中村改造和危旧简易楼群改造试点。东部新区：推进奥体文博中心区、汉峪总部经济区等城市综合体项目，加快高新区艺术中心建设。西部新区：加快核心区建设，建成省会文化艺术中心大剧院主体工程，完成市图书馆、群众艺术馆、美术馆主体结构施工和西客站场站一体化工程。滨河新区：继续抓好华山、泺口、徐李等片区开发项目，提升小清河综合治理水平。二是推进北跨和南控发展。加快长清黄河大桥、黄河公铁大桥、石济客运专线，以及商河温泉国际、澄波湖、鹊山龙湖建设，抓好化工产业园、济阳崔寨镇等北跨重要节点建设。深入实施南部山区生态功能区保护，以新型社区建设带动村庄整合，有序发展污染少、耗能低的文化旅游等产业。三是加强道路交通设施建设。加快城市轨道交通线网规划和新东站枢纽规划策划，做好济南至长清、济南至机场、济南绕城高速燕山立交至柳埠连接线、南绕城快速通道等项目前期工作。启动济南至乐陵高速公路及连接线、济南至东营公路建设工程。完善西部城区路网结构，推进二环西路地面道路和高架路工程建设。实施纬十二路、济齐路、刘长山路延长线等道路建设改造和30条道路大中修整治。采取综合措施，逐步解决交通拥堵问题。四是加强市政基础设施。抓好东区水厂一期、漏损管网、低压片区改造建设。推进供热计量改造，新建扩建腊山、唐冶和高新区供热工程，利用现有企业资源，开辟供热和治污新领域，建设城市燃气储备调峰基地。新建12个高、低压和特高压输变电工程项目。加快主城区污水全收集一期工程和水质净化三厂扩建配套建设。五是加强城市管理。建设市第三生活垃圾无害化处理厂、市生活废弃物处理中心和一批公厕、垃圾中转站。开展迎十艺节城市环境综合整治行动，实施景观照明、市容道路、铁路沿线、建筑渣土、重要经营场所、违法违章建设、城市出入口及窗口、城乡结合部、城区环境卫生、停车场整治十大工程，提高数字化城市管理水平，城市形象进一步改观。

　　(六)继续大力深化改革开放。改革开放是我市发展的强大动力，必须坚定不移地深入推进。一是文化体制改革。全面落实党的十七届六中全会精神，推动市属国有文艺院团转企改制。加强文化中介和行业组织建设，规范社会资本和外资进入文化领域的市场准入。培育大型骨干文化企业。二是医疗卫生体制改革。把村卫生室纳入基本药物制度实施范围，落实基本药物配备使用、医保报销和对乡村医生的补偿政策，推动新型农村合作医疗市级定点医疗机构住院即时结报、总额预付制等付费方式改革。三是国资国企改革。增强市四大投资集团融资能力，支持重点片区、重点工程开发建设，严格控制政府债务规模和风险。完善国有资本经营预算制度，推进国企开放性、市场化重组联合。四是农村改革。推进农村土地承包经营权股权化、集体资产股份化等农村产权资本化改革，加速农村土地流转经营。五是财税金融改革。全面实施政府非税收入执收成本核定等改革，推进社保基金预算编制改革，加强城建综合预算管理。推动农信社股份制、银行化改革，筹划组建市农村商业银行。六是资源性产品价格改革。推进环保收费制度改革，建立健全污染者付费制度，探索建立生态补偿制度和资源环境产权交易机制。

　　积极推进对外出口和经贸合作。一是大力促进外贸出口。实施出口市场多元化战略，稳定巩固传统市场，积极拓展欧美等容量大的市场和非洲、南美、东欧等有潜力的新兴市场，支持企业培育自主出口品牌，积极承接服务外包业务，增加机电、高新技术产品出口。完善企业服务平台，支持企业积极应对国际贸易摩擦。二是着力提高利用外资质量。重点在日韩、东南亚、欧洲等国家以及港澳台等地区精心策划招商活动，努力扩大利用外资规模。加强与国际知名中介机构、重要商会战略合作，吸引外资企业总部研发、营销、结算、物流中心等功能性机构落户我市。三是加强境外投资合作技术交流。做好海外工程承包、高端劳务输出、跨国公司培育等工作。促进航空、铁路、陆路、邮政口岸建设，加快济南综合保税区申建和明水国家级经济开发区的申报工作。

　　(七)大力推进创新型城市建设。把科技创新作为增强省会竞争力的关键，整合资源，培育主体，激发创新活力。一是加快创新平台体系建设。启动创新型县区试点，引导各类创新要素向企业聚集，争取新增5家国家级企业技术中心、工程技术研究中心和实验室，力争国家重大新药研发大平台一期工程竣工投用。加快科技成果转化和中介服务平台建设，加强重点行业、领域科技攻关和成果转化。加大知识产权战略实施力度，攻克一批具有自主知识产权的关键技术，抓好中创中间件参考平台等3个国家“核高基”项目，支持企业加强技术研发、实施重大科技专项，鼓励扶持首台重大装备研发生产，探索发电装备成套化模式。二是加强人才队伍建设。健全创新人才评价和激励机制，实施“5150引才倍增计划”和“百千万海内外人才引进工程”，引进扶持一批海内外领军人才及创业团队，建设多层次、梯队式创新人才队伍，把人才智力优势转化为创新优势、发展优势和竞争优势。深化科技体制改革，加快构建创新成果就地转化机制，支持企业与高校科研院所合作发展科技型中小企业，形成以企业为主体、市场为导向、产学研相结合的创新体系。三是加强企业家队伍建设。全社会都要关心和支持企业家干事创业，加大企业家培训力度，进一步提升境界，增强发展意识、创新意识和不甘落后、敢为人先的意识。鼓励支持想干事、能干事的企业家干大事、干成事，依法保护企业家合法权益，充分发挥他们的聪明才智，为济南发展作出更大贡献。鼓励国有企业认清形势、正视困难、抢抓机遇、积极作为，既要实现国有资产保值增值，又要通过改革创新振兴发展。

　　(八)加强民生和社会保障。坚持把改善民生作为促进发展的根本出发点和落脚点。今年继续提高财政支出投向民生和社会事业的比例。一是努力稳定和扩大就业。落实新一轮促进创业就业扶持政策，实施创业引领计划，抓好市级创业孵化基地建设。组织开展就业服务系列活动，促进高校毕业生、农业富余劳动力、城镇就业困难群体和退役人员就业，确保高校毕业生总体就业率稳定在80%以上，援助就业困难人员2万人，新增城镇就业10万人，农业富余劳动力转移就业5.2万人。二是完成社会保障任务。重点抓好非公有制经济组织从业人员、灵活就业人员、农村富余劳动力的参保工作，解决城镇未参保集体企业退休人员和被征地农民的养老保障问题，企业退休人员养老金标准平均增长10%以上。增加城乡居民低保、“五保”和优抚对象生活补助，农村低保标准每人每年达到2300元。建立工伤预防、补偿、康复一体化体系，提高工伤保险待遇水平。三是抓好保障性住房建设。积极引导社会力量参与保障性住房建设，完成开工建设1.8万套、竣工7500套保障性安居工程任务，适度集中建设农村住房。四是积极办好各级各类教育。实施县域义务教育均衡发展规划，推进学前教育三年行动计划，乡镇中心幼儿园规范率达到60%，严禁幼儿园以任何名义收取赞助费等与入园挂钩的费用。加快推进普通中小学标准化建设、中小学校舍安全、农村初中校舍改造等工程，加强校车安全和校园及周边治安工作，落实居住区教育设施配建工作。加大农村义务教育阶段困难学生资助力度，将农村初中和小学家庭困难寄宿学生生活费补助标准提高250元，分别达到1300元和1000元。五是加强公共文化服务工作。加快建设市民文化艺术中心和县级图书馆、文化馆等公共文化设施，动员社会力量建设各类博物馆20所以上。继续实施公益演出下基层、免费文化艺术辅导培训等文化惠民工程，向社会免费开放博物馆等公共文化服务设施和爱国主义教育基地。扎实做好十艺节筹备工作。六是强化基本医疗服务。深入实施基层机构卫生人员能力提升计划，加强重大和基本公共卫生服务项目督导检查，推行居民医保门诊统筹和生育津贴社会化发放，降低医疗个人负担费用，新型农村合作医疗筹资标准提高到人均300元。七是开展人口和计划生育“基层基础建设规范年”活动。推进流动人口基本公共服务均等化试点，稳定低生育水平。八是积极发展其他社会事业。广泛开展全民健身运动，建立养老信息系统，健全居家、社区、机构三位一体的养老服务模式。发展慈善事业，加快建立残疾人社会保障和服务体系，保障妇女儿童合法权益。加大科普力度，提高全民科学文化素质。今年政府工作围绕群众关注和反映强烈的问题作了一系列安排，同时确定了10件具体实事，要积极作为，抓好落实，确保兑现。

　　(九)强化节能减排和环境保护。突出抓好源头治理和长效机制建设，大力推进资源节约型和环境友好型社会建设。一是强化节能降耗。认真落实国家“十二五”节能减排综合工作方案，严格实施投资项目节能评估审查制度，完成3.0以下水泥磨机和建材行业747万吨落后产能淘汰任务，扶持山水水泥窑协同处置城市垃圾项目。优化能源结构，加快长清、平阴风电和力诺10兆瓦太阳能发电等项目建设，扩大新能源汽车等节能产品推广应用。抓好节约集约用地，逐步提高用地定额和投资产出强度控制标准，确保耕地保有量不少于36.6万公顷。二是强化环境保护。抓好火电、钢铁等重点行业脱硫、脱硝设施建设改造，开展新一轮重点流域排污企业限期治理，继续推进城区河道截污整治，完成遥墙、绣惠、相公等污水处理站建设，开工唐冶污水处理工程，推进工业企业废水深度治理。以工业废气、城市扬尘、机动车尾气为重点，开展扬尘污染防治分类挂牌管理，启动机动车环保定期检验和合格标志管理，将PM2.5纳入常规空气质量检测体系，提高空气环境质量改善率。三是推进生态城市建设。落实生态山东建设部署，扎实推进国家森林城市“十大工程”和水系生态“六大工程”建设，规划建设济西、白云湖、玫瑰湖、大沙河、清源湖等10大湿地公园保护恢复工程，实施绣源河风貌带等生态景观工程。增加西部新区和北跨新区的绿地、树木和水面面积，做好东部地区山林保护和破损山体修复工作。完成120平方公里水土流失治理和800个文明生态村建设，推进章丘农村环境连片整治重点示范工作。

　　(十)加强和创新社会管理。全面提高社会管理科学化水平，维护社会和谐稳定。一是推进社会管理创新“十大工程”和“民生政法”工程，建立人民调解、行政调解、仲裁调解、司法调解联动格局，健全基层矛盾纠纷调处机制，加强信访事项督查督办，做好法律服务和法律援助工作，在“事要解决”上狠抓落实。深入实施“六五”普法规划和依法治市纲要。创新社区管理办法，健全基层社会管理服务体系。二是积极开展文明城市创建活动，深入推进社会公德、职业道德、家庭美德、个人品德建设等群众性创建活动，支持社会工作者队伍和志愿者队伍建设，营造积极向上、文明健康的精神文化氛围。加强民族宗教和对台、侨务工作，做好双拥共建和民兵预备役工作。三是加强平安济南建设，完善社会治安打防管控一体化体系，严厉打击各类违法犯罪活动。建立重大事项风险评估机制，健全重大公共安全事件预警、应急、救援体系，发挥红十字会人道救助作用，加强现场救护和避险知识普及培训，提升城市防震减灾综合实力。防范金融风险，提升食品药品安全协调监管能力，打造农产品质量安全放心城市。强化安全生产责任制和重点领域专项整治，坚决遏制重特大安全事故的发生。

　　四、进一步加强政府自身建设

　　做好新一届政府工作，要理清思路，振奋精神，明确责任，提高效能，增强推动科学发展的能力，努力建设人民满意政府。

(一)振奋精神，争先进位。顺利完成今后五年的艰巨任务，在各地竞相发展中实现位次前移，绝非轻而易举的事情。在解决面临的诸多困难和问题中，首先要振奋精神、坚定信心、奋发图强、锐意进取。一是树立忧患意识，增强紧迫感。把济南的各项工作始终放在全国、全省的发展格局中定位，始终按照先进省会城市的标准衡量，对困难和问题保持清醒头脑，以时不我待、只争朝夕的精神，学习先进，缩小差距，开拓进取，争创一流。二是树立大局意识，增强使命感。把各级各部门工作始终放在全市大局中考虑，始终坚持局部利益服从整体利益，始终坚持围绕中心、服务大局，大力营造想干事、能干事、干成事的浓厚氛围，尽快掀起比效率、比业绩、比贡献的强劲热潮。三是树立敬业意识，增强责任感。各级政府工作人员要以对党和人民的事业高度负责的态度，恪尽职守，爱岗敬业，把心思用在谋发展上，把精力用在争先进上，面对事业充满激情，面对工作满腔热忱，面对挑战奋力拼搏，面对机遇抢抓不懈，始终保持不甘落后、昂扬向上的奋斗精神。

　　(二)增强能力，创新发展。面对复杂多变的经济形势和不断出现的新情况、新问题，必须加强能力建设。一是加强学习，增强综合素质和创新能力。建设学习型政府，认真学习理论知识，虚心学习先进经验，深入开展调查研究，提高按规律办事、创新发展的能力。加强战略性思考、前瞻性研究，精心谋划又好又快发展的思路办法。努力发挥省会优势，把优势资源和潜力变成优势产业和实力。二是用心做事，增强办事效率和破解难题的能力。坚持高标准、严要求，干工作精益求精，办事情提速提效。善于用发展的办法破解发展中的难题，用市场的手段推进市场经济的发展，用改革的精神探索改革创新的路子，用社会的力量解决社会面临的问题。三是依法行政，增强科学民主决策水平和执行能力。认真落实《山东省行政程序规定》，健全科学民主决策机制，完善专家咨询、论证、合法性审查、社会公示与听证等制度，对重大决策跟踪问效。严格行政执法程序，完善行政复议机制，进一步清理过时文件，精简行政审批事项，严格落实行政问责，建设阳光高效的法治政府。四是团结合作，增强沟通协作的主动性和发展合力。认真执行市人大及其常委会的决议决定，更加自觉地接受市人大、市政协及社会各界的监督和帮助，积极支持人大、政协、法院、检察院和各民主党派、工商联、无党派人士以及工会、共青团、妇联等更好地发挥作用。坚持问政于民、问计于民、问需于民，争取广大人民群众更多的理解和支持，充分调动各方面积极性和创造性，共同推进省会加快发展。

　　(三)求真务实，服务群众。强化宗旨意识、群众观念，始终保持与人民群众的血肉联系，把群众需要作为第一选择，真心为群众、为基层、为企业服好务，建设人民满意的服务政府。一是畅通渠道，健全机制。积极推进各级政务服务中心建设，发挥好12345市民服务热线、济南政府网、公共资源交易中心、行政审批中心等服务平台作用，健全联系群众和服务群众的工作机制。二是求真务实，多办实事。深入基层、深入群众，针对事关民生的难事、急事和基层、企业急需解决的问题，紧抓不放、及时解决。三是分级负责，狠抓落实。支持和督促各级政府、各部门积极主动地发挥好职能作用，明确办事规则和服务范围，不推诿、不扯皮、不刁难，多做雪中送炭的工作，多办攻坚克难的事情。加强绩效考核，把推动发展和为民服务的各项工作真正落到实处。

　　(四)艰苦奋斗，反腐倡廉。我市正处于改革发展的攻坚阶段，任务繁重而艰巨。各级领导和工作人员务必保持艰苦奋斗、廉洁勤政的作风，励精图治、埋头苦干。一是艰苦创业，真抓实干。尽快增强我市总体实力，实现争先进位，必须一个项目一个项目去抓、一个产业一个产业去推动、一件实事一件实事去落实，创业就要真抓实干，创业必须艰苦奋斗，以实干创造实绩，以实绩赢得民心。二是厉行节约，勤俭办事。坚持勤俭办一切事业，严格控制和压缩行政经费，精减会议和文件，专项治理公务用车，防止大手大脚、讲排场、乱花钱，建设节约型政府。三是廉洁从政，防治腐败。深入开展廉政建设和反腐败斗争，加快推进惩治和预防腐败体系建设。加强廉政风险防范管理，深入推进科技防腐，严格执行经济责任审计规定，完善领导干部制约监督机制。加大对审批权力运行、政府财政预决算、公共资源配置、重大建设项目审计监督检查力度，加强对中央和省市重大决策部署落实情况的监督检查，严厉查办违法违纪案件，建设廉洁政府。

　　各位代表！未来五年是我市建设更高水平小康社会、率先基本实现现代化的重要战略机遇期。让我们高举中国特色社会主义伟大旗帜，深入贯彻落实科学发展观，在省委、省政府和市委的坚强领导下，充分调动各方面的积极性和创造性，充分挖掘济南的优势和潜力，解放思想，抢抓机遇，团结奋斗，奋力拼搏，努力开创省会现代化建设的新局面！
